# Toward Personalized Tinnitus Treatment: An Exploratory Study Based on Internet Crowdsensing

**DOI:** 10.3389/fpubh.2019.00157

**Published:** 2019-06-25

**Authors:** Jorge Simoes, Patrick Neff, Stefan Schoisswohl, Jan Bulla, Martin Schecklmann, Steve Harrison, Markku Vesala, Berthold Langguth, Winfried Schlee

**Affiliations:** ^1^Department of Psychiatry and Psychotherapy, University of Regensburg, Regensburg, Germany; ^2^University Research Priority Program “Dynamics of Healthy Aging”, University of Zurich, Zurich, Switzerland; ^3^Department of Mathematics, University of Bergen, Bergen, Norway; ^4^Tinnitus Hub, Hemsworth, United Kingdom

**Keywords:** tinnitus, heterogeneity, crowdsensing, smart device, personalized treatment

## Abstract

**Introduction:** Chronic tinnitus is a condition estimated to affect 10–15% of the population. No treatment has shown efficacy in randomized clinical trials to reliably and effectively suppress the phantom perceptions, and little is known why patients react differently to the same treatments. Tinnitus heterogeneity may play a central role in treatment response, but no study has tried to capture tinnitus heterogeneity in terms of treatment response.

**Research Goals:** To test if the individualized treatment response can be predicted using personal, tinnitus, and treatment characteristics.

**Methods:** A survey conducted by the web platform Tinnitus Hub collected data of 5017 tinnitus bearers. The participants reported which treatments they tried and the outcome of the given treatment. Demographic and tinnitus characteristics, alongside with treatment duration were used as predictors of treatment outcomes in both an univariate as well as a multivariate regression setup. First, simple linear regressions were used with each of the 13 predictors on all of 25 treatment outcomes to predict how much variance could be explained by each predictor individually. Then, all 13 predictors were added together in the elastic net regression to predict treatment outcomes.

**Results:** Individual predictors from the linear regression models explained on average 2% of the variance of treatment outcome. “Duration of treatment” was the predictor that explained, on average, most of the variance, 6.8%. When combining all the predictors in the elastic net, the model could explain on average 16% of the deviance of treatment outcomes.

**Discussion:** By demonstrating that different aspects predict response to various treatments, our results support the notion that tinnitus heterogeneity influences the observed variability in treatment response. Moreover, the data suggest the potential of personalized tinnitus treatment based on demographic and clinical characteristics.

## 1. Introduction

Tinnitus is a condition characterized by an auditory perception, usually in the form of ringing or hissing, for which there is no corresponding external source (1). The prevalence of tinnitus has been estimated between 10 and 15% in the adult population (2, 3). From those, one fifth will require clinical intervention (4). Additionally, the mean annual cost of illness was estimated at 6.8 billion euros globally (5). On the individual level, tinnitus may be accompanied by comorbidities such as insomnia, anxiety and depression, constituting a high burden to patients (6). Current clinical guidelines recommend that clinicians target those potential comorbidities, and although no treatment has shown efficacy in randomized clinical trials to reliably and effectively suppress the phantom perceptions, it is clear that various treatment options result in different degree of improvements—most likely because of the underlying heterogeneity of the etiology and pathophysiology of tinnitus (2, 7, 8). The clinical guidelines also recommend different management strategies for tinnitus, including, but not limited to, psycho-education, counseling, cognitive behavior therapy, hearing aids when assessed as necessary and sound therapy (2). Importantly, the current clinical understanding is that certain treatments may not be suitable/effective for all, and clinicians should recommend treatments to patients in an individual basis (8). Thus, albeit the low evidence levels for treatments on a group level, these same treatments may be beneficial in specific cases on the individual level.

From a clinical perspective, bothersome, chronic, and subjective tinnitus is a common and challenging form of tinnitus (2, 6). However, this form of tinnitus might be highly heterogeneous. In recent years, the notion of tinnitus as a complex, multi-faceted condition gained traction (9). For that reason, researchers and clinicians have drawn their attention to the different ways of tinnitus manifestation, including its etiology (e.g., sound blast, persistent loud noise exposure, whiplash, etc.), phenotype (e.g., type of sound perceived, laterality of the sound perception, presence of hearing loss, etc.), and accompanying comorbidities (e.g., insomnia, depression, anxiety, etc.). Such heterogeneity constitutes a complex puzzle that challenges both researchers and clinicians in their understanding of the pathophysiology of tinnitus and in the development of new treatments (1). Importantly, tinnitus heterogeneity may account for the low success rates of clinical trials at the group level, as well as why certain individuals respond positively to specific treatments (8, 10).

Noteworthy efforts to capture tinnitus heterogeneity include the studies from Langguth et al. (11), Tyler et al. (12) and Van den Berge et al. (13). Overall, the studies showed modest results without a clear delineation of tinnitus subtypes. However, those studies were limited due to sample size and/or homogeneous samples recruited from specialized tinnitus clinics. It is yet unclear how representative samples from tertiary clinics represent the whole tinnitus population; thus, we consider a broader data sample necessary to capture a yet unexplored facet of tinnitus heterogeneity (14).

Crowdsourced health research studies have been proposed as a mean to circumvent the difficulties experienced during patient's recruitment, such as the increased costs of adding participants to a study and the homogeneous sample representation from tertiary clinics (15). Crowdsourcing can be defined as the collaborative collection of data in which individuals and/or institutions participate voluntarily (15, 16). When the data is collected through mobile devices, such as smartphones, tablets, or wearable devices, the term crowdsensing is commonly used (17). The number of policy makers, health providers and academics using such technologies increased drastically in the last decade due to the ubiquity of mobile and sensing devices (18). Especially in tinnitus research, crowdsensing has been substantially used (14, 17, 19, 20). Importantly, such technologies may yield new insights about phenomena hardly accessible to traditional settings.

To the best of our knowledge, no study tried to capture tinnitus' heterogeneity using crowdsensing technology, especially in terms of treatment response. Our study aims to fill that research gap. We collected crowdsensed data from an online tinnitus self-help platform to explore tinnitus heterogeneity avoiding the aforementioned limitations during data collection, namely the reduced sample size and/or homogeneous patient representation. First, we investigated whether tinnitus heterogeneity could be expressed not only in terms of phenotype, etiology and comorbidities as has previously been done, but also in terms of treatment response. To investigate this hypothesis, we modeled each predictor (i.e., tinnitus characteristics and demographics) individually as an independent variable on single linear regressions with treatment outcomes for 25 different treatments as dependent variables. Second, we investigated whether tinnitus heterogeneity could predict treatment response from demographic factors and tinnitus characteristics. We operationalized this hypothesis by combining all predictors in a statistical model to predict the outcome of treatments.

## 2. Methods

Data for our sample were collected by Tinnitus Hub. Founded in 2015 by SH and MV, the Tinnitus Hub operates “Tinnitus Talk” (www.tinnitustalk.com), created in 2011, the largest online, anglophone self-help platform for tinnitus patients. The survey took place between February 8th and March 13th of 2016. Members of the forum received a link to the digital survey. We collected information of 5017 participants, from those 2916 reported trying at least one treatment and thus were included in the data set for the final analysis. It was not possible to obtain written informed consent from the users of Tinnitus Talk, but the “Terms and Rules” of the website informed the users that the collected data will be analyzed for scientific purposes. All the data were saved anonymously. A similar dataset was used in a former study (14).

Personal and tinnitus information was collected from participants of the survey alongside questions about which tinnitus-related treatments were tried and were used as independent values in our statistical models. In total, 13 factors were included in our analysis (**Table 2**). Additionally, participants were asked to rate how effective a given treatment was in reducing the distress and/or suppressing the noise perception, and the duration of the treatment retrospectively (1: “this treatment made my tinnitus much worse,” 2: “this treatment made my tinnitus mildly worse,” 3: “this treatment had no effect on my tinnitus,” 4: “this treatment made my tinnitus slightly better,” and 5: “this treatment made my tinnitus much better”).

Our analysis included the outcome of 25 different treatments and used as dependent variables in our statistical model. Participants consented to have their anonymous data used for scientific research. Simple linear regressions were performed for individual predictors (i.e., demographics and tinnitus characteristics, and treatment duration) on treatment outcomes (i.e., dependent variable). Regressions were weighted based on the number of treatments that patients tried and *p*-values were adjusted for multiple comparisons using Hommel correction (21, 22). Collinearity was assessed with the variance inflation factor (VIF). The VIF is the ratio of variance in a model with multiple predictors, divided by the variance of a model with one predictor alone (23). The high VIF values in our models indicated that models containing all 13 demographic factors and tinnitus characteristics as predictors would contain high collinearity. To address this issue, we used elastic net regularization (24). Elastic net accounts for collinearity by penalizing the coefficients in the model either by shrinking their values or by setting them to 0 (24). We ran a n-fold cross validated elastic net to estimate the optimal lambda (i.e., one of the penalizing coefficients from elastic net) over 11 different alpha values ranging from 0 (i.e., RIDGE regression) to 1 (i.e., Lasso Regression). For this analysis, the predictors encoded as factors were converted into dummy variables as a prerequisite from the statistical software. We selected the models with minimized mean squared error for our final analysis.

All statistical analysis was conducted with R statistical software (25), alongside the “tidyverse” package (26). Power analysis were calculated using the "effsize" package (27) and the elastic net was performed by the "GLMnet" package (24). Non-parametric tests were used when statistical assumptions of parametric tests were not met. *P*-values below 0.05 were considered statistically significant.

## 3. Results

**Figure 1:**
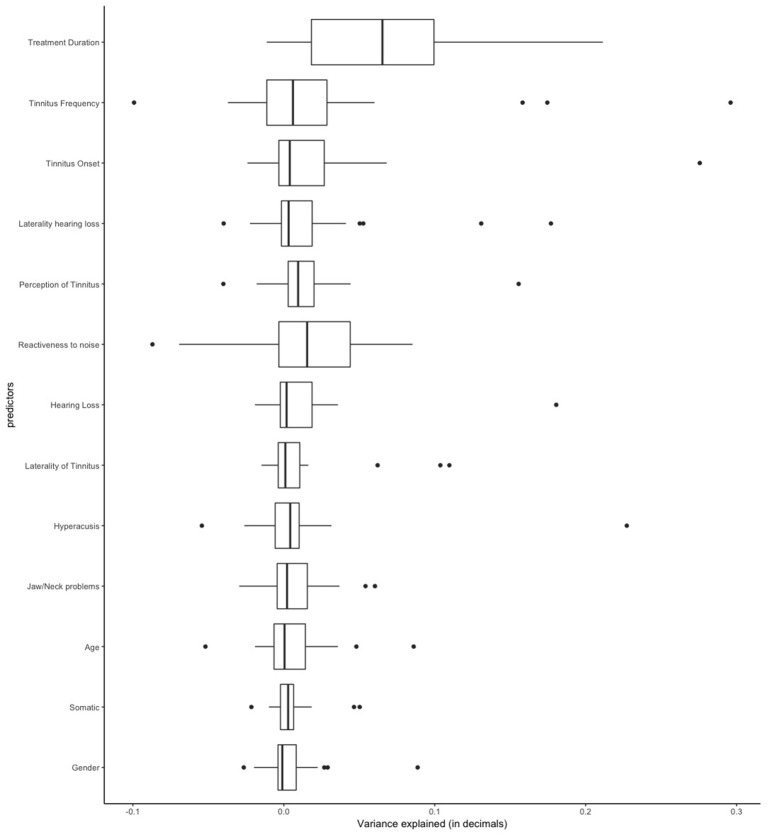
Amount of variance explained in the linear regression models by each predictor across all different 25 treatments.

**Figure 2:**
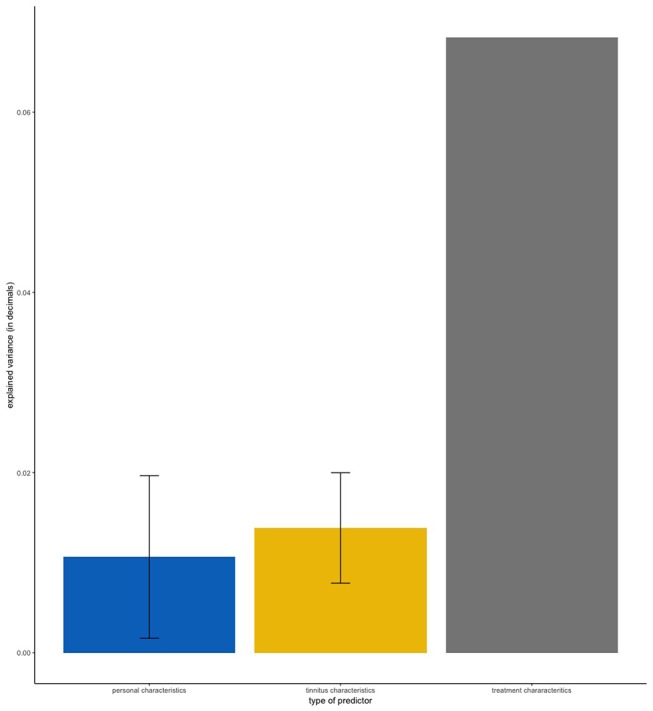
Mean amount of variance explained by type of predictor. Error bars represent standard deviation. “Personal Characteristics” contains the predictors Age, Gender, and Tinnitus Onset. “Tinnitus Characteristics” contains the predictors Tinnitus Frequency, Laterality of Hearing Loss, Perception of Tinnitus, Reactiveness to Noise, Hearing Loss, Laterality of Tinnitus, Hyperacusis, and Jaw/Neck Problems. “Treatment Characteristics” contains the predictor Treatment Duration.

Table 1 shows the frequency of each treatment in our sample. Clinical and demographic characteristics of the sample are summarized in Table 2. First, we applied linear regression models with individual predictors as independent variables on the self-reported treatment outcomes as dependent variables. The aim of this analysis was to test how much variance could be explained by individual predictors for the different treatments. Figure 1 shows the average amount of variance explained by each predictor on all 25 different treatments. A summary of all statistical models can be found in the Supplementary Materials. The amount of variance explained by single predictors over all treatments was 2% on average. Next, we investigated what type of predictor could explain most of the variance of treatment outcomes. For this analysis, we grouped predictors in three groups: personal, tinnitus and treatment characteristics (Figure 2). Personal and tinnitus characteristics could explain, on average, the same amount of variance.

**Table 1:** Sample size of each treatment.

**Treatment**	***n***
Self Sound Stimulation	1,562
Supplements and Herbal	1,157
Antidepressants	785
Hearing Aid	681
Acunpuncture	621
Masker	503
Chiropractor	489
Homeopathic	425
Psychologist	388
Cognitive Behavior Therapist	371
Tinnitus Retraining Therapy	370
Steroids	346
Off-label Medication	312
Psychiatrist	298
Neurofeedback / Meditation	270
Books / self help	254
Gabaergic medication	237
Notched Music	223
Soundcure	144
Acoustic Neuromodulation	120
Neuromonics	95
Low Level Laser Therapy	65
Retigabbine	53
Hyperbaric Oxygen Therapy	46
Transcranial Magnetic Stim.	45

**Table 2:** Sample's demographic and tinnitus characteristics.

**Predictor**	**Levels**	***n***	**Percentage**
Gender	Male	1,712	58.8%
	Female	1,181	40.5%
	Other	21	0.7%
Age	Under 18	13	0.4%
	18–24	162	5.6%
	25–34	364	12.5%
	35–44	427	14.7%
	45–54	606	20.8%
	55–64	869	29.8%
	65–74	405	13.9%
	75 +	58	2.0%
	Prefer not to say	10	0.3%
Tinnitus onset	Less than 3 months	147	5.0%
	4–6 months	156	5.4%
	6–12 months	293	10.1%
	1–2 years	427	14.7%
	2–3 years	359	12.3%
	3–5 years	347	11.9%
	5–10 years	388	13.3
	10–20 years	339	11.6%
	20 + years	458	15.7%
Noise reactiveness	Sounds have no affect	587	20.1%
	Some sounds make it a lot worse	627	21.5%
	Some sounds make it somewhat worse	354	12.1%
	Some sounds make it better and some make it worse	725	24.9%
	Some sounds make it somewhat better	212	7.3%
	Some sounds make it a lot better	113	3.9%
	NA	296	10.2%
Hyperacusis	No	1,006	34.5%
	Mildly	795	27.3%
	Moderately	776	26.6%
	Severely	291	10.0%
	NA	96	3.3%
Somatic	No	1,643	56.4%
	Yes	1,056	36.2%
	NA	215	7.4%
Jaw and neck problems	Problems with Jaw	261	9.0%
	Problems with Neck	503	17.3%
	Problems with Jaw and Neck	407	14.0%
	NA	1,743	59.8%
Hearing loss	Mild hearing Loss	1,265	43.4%
	Moderate hearing loss	400	13.7%
	Severe hearing loss	152	5.2%
	NA	1,097	37.6%
Laterality of hearing loss	Both ears	699	24.0%
	One ear	1,119	38.4%
	NA	1,096	37.6%
Tinnitus frequency	Low (<1 kHz)	152	5.2%
	Mid (1–3kHz)	151	5.2%
	Mid high (3–8 kHz)	525	18.0%
	Very high (8 kHz +)	350	12.0%
	Several dis in Hearing	77	2.6%
	Unsure	563	19.3%
	Na	1,096	37.6%
Perception of tinnitus	One ear	688	23.6 %
	Both ears	1,031	35.4 %
	More in the brain	204	7 %
	In the ears and brain	952	32.6 %
	Not sure	39	1.3 %
Perception of tinnitus during the day	Does not change at all	774	26.6 %
	Fluctuates, no pattern	1,369	46.9 %
	Fluctuates, better in the morning	131	4.5 %
	Fluctuates, better in the evening	626	21.4 %
	NA	14	0.4 %

**Figure 3:**
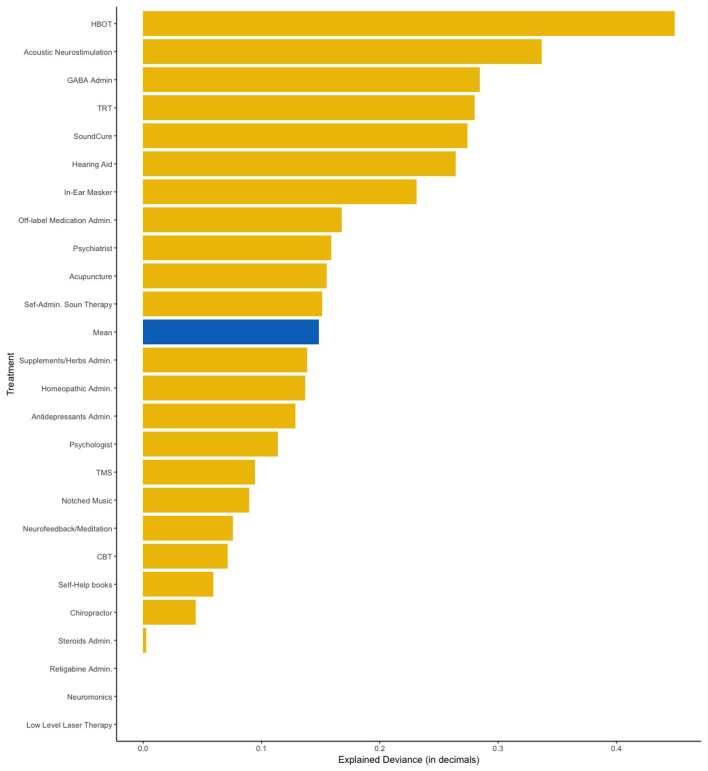
Amount of variance explained by the Elastic Net model with all the 13 predictors added simultaneously. HBOT, Hyperbaric Oxygen Therapy; TRT, Tinnitus Retraining Therapy; TMS, Transcranial Magnetic Stimulation; CBT, Cognitive Behavior Therapy.

As shown in Figures 1, 2, the predictor “Duration of Treatment” explained on average more variance than the remaining predictors (*p* < 0.05). To further explore the relationship between treatment duration and treatment outcome, we clustered the average treatment outcomes based on their duration. The results can be found in Figure 3, where our analysis of variance showed no trend of time over treatment outcome (*p* = 0.99).

Next, we fitted all predictors as independent variables and self-reported treatment outcomes as the dependent variable in our elastic net regression model. This analysis aimed to measure how much of the deviance on treatment outcomes can be explained by combining all analyzed items. Figure 3 shows the amount of deviance explained by all predictors for each of the 25 treatments. On average, 16% of the deviance could be explained by all predictors combined. Table 3 summarizes which predictors were considered statistically significant by the elastic net and linear regressions respectively.

**Table 3:** Predictors identified as significant by the elastic net model (X) and by linear regressions (O).

	**Duration of treatment**	**Laterality of hearing loss**	**Fluctuation of sound perception**	**Noise reactiveness**	**Jaw/Neck problems**	**Onset**	**Age**	**Hyperacusis**	**Gender**	**Tinnitus frequency**	**Hearing loss**	**Somatic**	**Laterality of tinnitus**
Acoustic Neuromodulation	X	X	X	X	X	X	X	X	X	X	X		
Hearing aid	X/O	X/O	X	X/O	X/O	X/O	X	X	X	X	X/O		O
Self Admin. Sound Therapy	X/O	X	X/O	X/O	X	X	X	X	X/O	X			
TRT	X/O	X	X	X	X	X	X/O	X		X	X/O		
Antidepressants	X/O	X	X/O	X	X	X	X/O	X/O		X			
Soundcure	X/O	X	X	X		X	X	X	X	X/O			
Psychiatrist	X	X	X	X		X	X	X	X		X		
Psychologist	X	X	X/O	X		X	X	X	X		X		
Supplements/Herbal admin.	X	X	X	X	X		X			X	X		
Homeopathic admin.	X	X	X	X	X	X	X		X				
GABA admin.	X/O			O	X/O		X/O	X		X	X		
In ear masker	X/O	X	X	O				X		X	X		
Acunpuncture	X	X	X		X/O	X	X		X/O				
Hyperbaxic Oxygen Therapy	X	X			X	X		X		X			
Notched music	X/O	X/O	X	X					X				
Off Label Medication admin.	X		X	X	X	X							
Self learning	X	X		X	X	X							
CBT	X		X/O					X	O				
Chiropractor	X				X								
Neurofeedback	X/O						X						
Steroids admin.					X	X							
LowLevelLaser Therapy	X												
Neuromonics	X												
Retigabine admin.		X											
Transcranianl Magnetic Stim.		X											

*Coefficients associated with significant predictors can be found in Supplementary Materials*.

**Figure 4:**
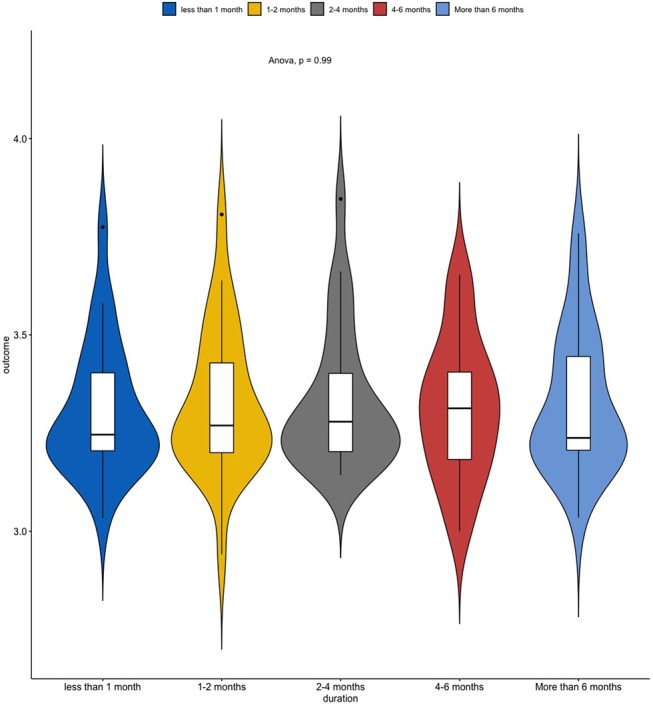
Mean treatment outcomes on a 1–5 Likert scale clustered by treatment duration.

Lastly, we conducted one exploratory analysis based on the coefficients obtained by both models to identify clinical markers of treatment success. From coefficients estimated by linear regression, we observed that participants who reported responding positively to sounds (i.e., rating a 4 or 5 in the Likert scale) reported more frequently benefiting positively to treatments with an acoustic component. Thus, we subset only patients who reacted positively to sounds and divided treatments with and without an acoustic component (Figure 4). Our group mean comparison analysis corroborated our data-driven hypothesis, as patients who reported reacting positively to sounds also reported higher outcomes with treatments with an acoustic component (*p* = 0.02, Cohen's d = 1.07).

## 4. Discussion

In this study we investigated whether personal, tinnitus, and treatment characteristics collected from an internet self-help platform population can be used to explain which patients are responding to different treatments. Similar attempts to predict treatment outcomes with patients' characteristics have been tried in a spectrum of mental conditions, including lower back pain (28), depression (29), post traumatic stress disorder (30), obsessive-compulsive disorder (31), substance abuse (32), and tinnitus itself (33). To the best of our knowledge, this is the first study attempting to capture tinnitus' heterogeneity in terms of a wide range of treatment responses using crowdsensing technology. Moreover, whereas most studies tried to predict the outcome of a single treatment, our study aimed to predict the outcome of 25 different treatments.

Our results showed that 2% of the variance of treatment outcomes could be explained, on average, by individual predictors (Figure 2). Additionally, our analysis showed that both personal characteristics and tinnitus characteristics, despite being significant predictors for multiple treatments (Table 3), could explain little variance on average. At first glance, it seems that the analyzed parameters have only a small impact on treatment outcome, but the average amount of deviance explained by the elastic net combining all 13 predictors into a single model was 16%, after accounting for covariance. We identified multiple statistically significant predictors in both regression setups (Table 3), but the individual amount of variance they could explain was limited. These results suggest that although no single predictor is paramount to predict the treatment outcomes, personal, tinnitus, and treatment characteristics may have a predictive role when combined. Altogether, those characteristics could be used in the future to predict treatment responsiveness in tinnitus, especially after better markers of treatment success are identified. For instance, our analysis did not include information about patients' personality, depression or tinnitus-related distress, nor did it collect information of the sequence in which treatments were tried or whether treatments were tried simultaneously.

Capturing tinnitus heterogeneity has been proposed as an important clinical and scientific goal, but previous attempts obtained limited results (12, 13). Importantly, tinnitus heterogeneity may explain why only a subset of patients are responding to specific treatments (10). A broader comprehension of tinnitus, encompassing not only demographics and tinnitus characteristics, but also treatment response, could, for example, explain the limited treatment efficacy seen in clinical practice (2). For instance, it is yet unclear whether previous successful or unsuccessful treatments have any predictive power on the outcomes of future treatments. Ultimately, the subtyping of tinnitus could lead to personalized care, a long-standing request by both clinicians and patients (6). Our results, though modest, suggest that personalized treatment for tinnitus patients based on patients' personal, tinnitus, and treatment characteristics should be feasible.

**Figure 5:**
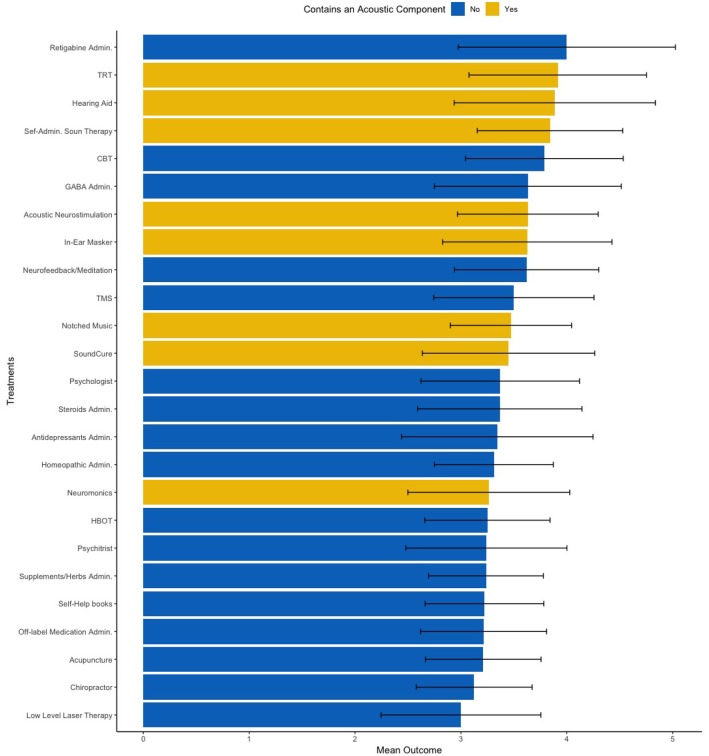
Mean treatment outcomes on a 1–5 Likert scale clustered by treatments with an acoustic component (yellow) and without an acoustic component (blue). Error bar accounts for the standard deviation across all 25 treatments. TRT, Tinnitus Retraining Therapy; CBT, Cognitive Behavior Therapy; TMS, Transcranial Magnetic Stimulation; HBOT, Hyperbaric Oxygen Therapy.

One example of future implications that this type of analysis could lead to, is the effect of noise reactiveness in the outcomes of treatments with and without an acoustic component (Figure 5). Our results suggest that participants whose tinnitus respond positively to sounds tend to benefit more from treatments with an acoustic component than from treatment without such component. Although future studies should try to replicate these results, we believe that the insights from large data sets such as these could have meaningful effects in tinnitus care and research. For instance, such insights could help researchers define new, fine-grained inclusion criteria for future clinical trials in acoustic-based treatments.

Regarding treatment duration, the predictor that could, on average, explain most of the variance, did not show any statistically significant difference between time periods. These results should be interpreted with caution as it is well-known that certain treatments, such as cochlear implants, require some time for adaptation whereas other treatments, such as antidepressants, require longer periods to be effective. Nonetheless, our results support the notion that the duration of treatment is not inherently beneficial or detrimental to the treatment's efficacy.

Our study comes with some inherent limitations. First, we did not have access to information about treatments which were performed in an overlapping span of time, thus we were unable to account for possible interaction between treatments. Second, our outcome measure was retrospective and subjective, which could have biased the results. We consider a subjective metric, although coarser than an objective one such and the Tinnitus Handicap Inventory, adequate for this type of analysis given the multiple treatments that a single patient tried and the sometimes-long period of time between the administration of a treatment and the survey. Nevertheless, further prospective studies analyzing outcome predictors would be desirable. Third, although we examined 25 different treatments, this number was insufficient to capture the whole complexity of available interventions for tinnitus treatments. Cognitive Behavior Therapy (CBT), for example, can be performed in a span of days or months, sessions can be individual or in group, a wide range of techniques can be applied in each session, etc. Such variety of treatment details and subtypes were not exclusive to CBT, but rather a commonality across treatments. Fourth, we chose a limited number of potential predictors for the survey, but we might have missed other important items. Particularly we would expect that there may exist further items that may be relevant for response to some of the investigated treatments. Finally we are aware that the investigated sample, albeit large and international, might not be representative of all patients with tinnitus (14).

## 5. Conclusion

Our results suggest that tinnitus heterogeneity could be expressed in terms of treatment response. The variance explained by individual predictors on treatment outcomes suggests that specific traits could explain why certain people are responding positively to a given treatment. In the future, especially with the availability of “big” multi-faceted data, a better understanding of the factors involved in treatment responsiveness could lead to individualized, optimal tinnitus management.

## Ethics Statement

The data set was collected in 2016 through a survey in the tinnitus hub online forum (https://www.tinnitushub.com), and was shared to the authors.

## Author Contributions

JS wrote the manuscript. JS, WS, and JB defined the study design and interpreted the results. PN, MS, SS, and BL interpreted the results, and provided critical feedback during the review. SH and MV were responsible for data collection.

### Conflict of Interest Statement

The authors declare that the research was conducted in the absence of any commercial or financial relationships that could be construed as a potential conflict of interest.
