# Supplementary material for: Toward Personalized Tinnitus Treatment: An Exploratory Study Based on Internet Crowdsensing
Source: Front Public Health. 2019 Jun 25;7:157. doi: 10.3389/fpubh.2019.00157 (PMC6604754; doi:10.3389/fpubh.2019.00157)
Supplement: Supplementary file 1 [file Data_Sheet_1.PDF]

Supplementary Material: Towards personalized Tinnitus Treatment: An Exploratory Study Based on Internet Crowdsensing

1 SUPPLEMENTARY DATA

Supplementary Material should be uploaded separately on submission. Please include any supplementary data, figures and/or tables.

Supplementary material is not typeset so please ensure that all information is clearly presented, the appropriate caption is included in the file and not in the manuscript, and that the style conforms to the rest of the article.

2 SUPPLEMENTARY TABLES AND FIGURES

Acoustic Neuromodulation

	r.squared	adj.r.squared	sigma	statistic	p.value	Adjusted p.value	df
Gender	0.017	0.001	1.498	1.033	0.359	0.633	3
Age	0.071	0.022	1.482	1.440	0.206	0.633	7
Onset	0.113	0.049	1.461	1.762	0.092	0.598	9
Noise reactivness	0.096	0.048	1.462	1.998	0.072	0.574	7
Hyperacusis	0.028	-0.006	1.502	0.836	0.505	0.633	5
Somatic	0.012	-0.005	1.502	0.684	0.507	0.633	3
Jaw/neck problems	0.078	0.054	1.457	3.269	0.024	0.253	4
Hearing loss	0.024	-0.001	1.499	0.962	0.413	0.633	4
Laterality of Hearing loss	0.056	0.042	1.520	3.861	0.054	0.430	2
Frequency of Tinnitus	0.054	-0.024	1.571	0.690	0.633	0.633	6
Laterality of Tinnitus	0.028	-0.006	1.503	0.823	0.513	0.633	5
Fluctuation of Tinnitus	0.062	0.037	1.470	2.542	0.060	0.478	4
Duration of treatment	0.080	0.039	0.652	1.974	0.088	0.598	6
Sum	-	0.249	-	-	-	-	-

Table S1. summary of statistical models with the outcomes of the treatment ”Acoustic Neuromodulation” as dependent variable

Acupuncture							
	r.squared	adj.r.squared	sigma	statistic	p.value	Adjusted p.value	df
Gender	0.023	0.018	1.283	4.792	0.003	0.029	4
Age	0.005	-0.008	1.300	0.386	0.928	0.928	9
Onset	0.017	0.004	1.292	1.311	0.235	0.928	9
Noise reactivness	0.003	-0.007	1.299	0.322	0.925	0.928	7
Hyperacusis	0.012	0.005	1.291	1.851	0.117	0.780	5
Somatic	0.002	-0.001	1.296	0.594	0.552	0.928	3
Jaw/neck problems	0.029	0.025	1.279	6.217	0.000	0.004	4
Hearing loss	0.007	0.002	1.293	1.446	0.228	0.928	4
Laterality of Hearing loss	0.002	-0.001	1.277	0.777	0.379	0.928	2
Frequency of Tinnitus	0.010	-0.003	1.279	0.791	0.557	0.928	6
Laterality of Tinnitus	0.012	0.005	1.291	1.806	0.126	0.780	5
Fluctuation of Tinnitus	0.009	0.005	1.293	1.952	0.120	0.780	4
Duration of treatment	0.087	0.079	0.524	11.651	0.087	0.627	6
Sum	-	0.124	-	-	-	-	-

Table S2. summary of statistical models with the outcomes of the treatment ”Acupuncture” as dependent variable

Antidepressants							
	r.squared	adj.r.squared	sigma	statistic	p.value	Adjusted p.value	df
Gender	0.001	-0.003	2.274	0.277	0.842	0.962	4
Age	0.031	0.021	2.247	3.067	0.002	0.021	9
Onset	0.022	0.012	2.257	2.197	0.026	0.180	9
Noise reactivness	0.014	0.006	2.263	1.852	0.087	0.346	7
Hyperacusis	0.022	0.017	2.251	4.357	0.002	0.017	5
Somatic	0.006	0.004	2.266	2.528	0.080	0.322	3
Jaw/neck problems	0.008	0.004	2.266	1.995	0.113	0.453	4
Hearing loss	0.001	-0.002	2.273	0.387	0.763	0.962	4
Laterality of Hearing loss	0.000	-0.002	2.218	0.002	0.962	0.962	2
Frequency of Tinnitus	0.032	0.022	2.191	3.247	0.007	0.061	6
Laterality of Tinnitus	0.014	0.009	2.260	2.833	0.024	0.166	5
Fluctuation of Tinnitus	0.022	0.018	2.255	5.693	0.001	0.008	4
Duration of treatment	0.063	0.057	0.878	10.485	0.000	0.000	6
Sum	-	0.163	-	-	-	-	-

Table S3. summary of statistical models with the outcomes of the treatment ”Antidepresssants” as dependent variable

CBT							
	r.squared	adj.r.squared	sigma	statistic	p.value	Adjusted p.value	df
Gender	0.037	0.029	1.860	4.683	0.003	0.035	4
Age	0.019	0.000	1.887	1.003	0.429	0.961	8
Onset	0.007	-0.015	1.901	0.312	0.961	0.961	9
Noise reactivness	0.011	-0.005	1.892	0.667	0.676	0.961	7
Hyperacusis	0.021	0.010	1.877	1.969	0.099	0.690	5
Somatic	0.007	0.002	1.885	1.380	0.253	0.902	3
Jaw/neck problems	0.007	-0.002	1.889	0.809	0.489	0.961	4
Hearing loss	0.006	-0.002	1.889	0.777	0.507	0.961	4
Laterality of Hearing loss	0.008	0.003	1.729	1.707	0.193	0.845	2
Frequency of Tinnitus	0.051	0.029	1.706	2.319	0.045	0.445	6
Laterality of Tinnitus	0.011	0.000	1.887	1.002	0.407	0.961	5
Fluctuation of Tinnitus	0.037	0.029	1.860	4.684	0.003	0.035	4
Duration of treatment	0.074	0.061	0.721	5.796	0.074	0.652	6
Sum	-	0.140	-	-	-	-	-

Table S4. summary of statistical models with the outcomes of the treatment ”Cognitive Behavior Therapy” as dependent variable

Chiropractor

	r.squared	adj.r.squared	sigma	statistic	p.value	Adjusted p.value	df
Gender	0.001	-0.005	1.336	0.179	0.911	0.911	4
Age	0.008	-0.007	1.337	0.539	0.805	0.911	8
Onset	0.017	0.001	1.332	1.048	0.399	0.911	9
Noise reactivness	0.030	0.018	1.321	2.449	0.024	0.266	7
Hyperacusis	0.003	-0.005	1.336	0.406	0.804	0.911	5
Somatic	0.010	0.006	1.329	2.410	0.091	0.818	3
Jaw/neck problems	0.019	0.013	1.324	3.162	0.024	0.268	4
Hearing loss	0.002	-0.005	1.336	0.264	0.852	0.911	4
Laterality of Hearing loss	0.002	-0.002	1.316	0.528	0.468	0.911	2
Frequency of Tinnitus	0.023	0.007	1.310	1.388	0.229	0.911	6
Laterality of Tinnitus	0.004	-0.004	1.335	0.461	0.764	0.911	5
Fluctuation of Tinnitus	0.014	0.008	1.324	2.308	0.076	0.682	4
Duration of treatment	0.061	0.051	0.533	6.255	0.000	0.000	6
Sum	-	0.076	-	-	-	-	-

Table S5. summary of statistical models with the outcomes of the treatment ”Chiropractor” as dependent variable

Self-help book

	r.squared	adj.r.squared	sigma	statistic	p.value	Adjusted p.value	df
Gender	0.005	-0.007	1.349	0.452	0.716	0.901	4
Age	0.017	-0.006	1.349	0.731	0.625	0.901	7
Onset	0.049	0.018	1.333	1.577	0.132	0.836	9
Noise reactivness	0.020	-0.003	1.347	0.861	0.524	0.901	7
Hyperacusis	0.024	0.008	1.339	1.516	0.198	0.895	5
Somatic	0.026	0.018	1.333	3.331	0.037	0.411	3
Jaw/neck problems	0.002	-0.010	1.351	0.194	0.901	0.901	4
Hearing loss	0.023	0.012	1.337	1.995	0.115	0.807	4
Laterality of Hearing loss	0.019	0.013	1.233	2.967	0.087	0.643	2
Frequency of Tinnitus	0.044	0.012	1.234	1.364	0.241	0.901	6
Laterality of Tinnitus	0.024	0.008	1.340	1.506	0.201	0.895	5
Fluctuation of Tinnitus	0.011	-0.001	1.346	0.908	0.438	0.901	4
Duration of treatment	0.052	0.033	0.551	2.743	0.020	0.243	6
Sum	-	0.094	-	-	-	-	-

Table S6. summary of statistical models with the outcomes of the treatment ”Self-Help books” as dependent variable

GABA

	r.squared	adj.r.squared	sigma	statistic	p.value	Adjusted p.value	df
Gender	0.005	-0.004	2.419	0.531	0.589	0.589	3
Age	0.113	0.086	2.308	4.167	0.000	0.003	8
Onset	0.068	0.036	2.371	2.088	0.038	0.227	9
Noise reactivness	0.081	0.057	2.345	3.356	0.003	0.031	7
Hyperacusis	0.042	0.026	2.383	2.547	0.040	0.241	5
Somatic	0.012	0.003	2.410	1.375	0.255	0.589	3
Jaw/neck problems	0.071	0.059	2.341	5.971	0.001	0.007	4
Hearing loss	0.016	0.004	2.409	1.294	0.277	0.589	4
Laterality of Hearing loss	0.057	0.051	2.459	8.454	0.004	0.038	2
Frequency of Tinnitus	0.080	0.046	2.465	2.350	0.044	0.265	6
Laterality of Tinnitus	0.030	0.013	2.398	1.806	0.128	0.514	5
Fluctuation of Tinnitus	0.013	0.000	2.414	1.000	0.394	0.589	4
Duration of treatment	0.133	0.115	0.830	7.113	0.000	0.000	6
Sum	-	0.491	-	-	-	-	-

Table S7. summary of statistical models with the outcomes of the treatment ”GABAergic medication” as dependent variable

Hearing Aid

	r.squared	adj.r.squared	sigma	statistic	p.value	Adjusted p.value	df
Gender	0.008	0.003	2.077	1.762	0.153	0.613	4
Age	0.014	0.003	2.077	1.318	0.239	0.820	8
Onset	0.038	0.026	2.053	3.273	0.001	0.011	9
Noise reactivness	0.027	0.019	2.061	3.142	0.005	0.038	7
Hyperacusis	0.015	0.010	2.070	2.631	0.033	0.234	5
Somatic	0.007	0.005	2.076	2.561	0.078	0.459	3
Jaw/neck problems	0.041	0.037	2.042	9.676	0.000	0.000	4
Hearing loss	0.040	0.036	2.043	9.441	0.000	0.000	4
Laterality of Hearing loss	0.000	-0.001	2.007	0.052	0.820	0.820	2
Frequency of Tinnitus	0.007	-0.001	2.006	0.877	0.496	0.820	6
Laterality of Tinnitus	0.022	0.017	2.063	3.890	0.004	0.031	5
Fluctuation of Tinnitus	0.002	-0.003	2.082	0.376	0.770	0.820	4
Duration of treatment	0.217	0.211	0.845	37.340	0.000	0.000	6
Sum	-	0.360	-	-	-	-	-

Table S8. summary of statistical models with the outcomes of the treatment ”Hearing Aid” as dependent variable

Homeopathics

	r.squared	adj.r.squared	sigma	statistic	p.value	Adjusted p.value	df
Gender	0.022	0.015	1.264	3.113	0.026	0.236	4
Age	0.036	0.020	1.261	2.209	0.033	0.294	8
Onset	0.019	-0.000	1.273	0.985	0.447	0.909	9
Noise reactivness	0.057	0.043	1.245	4.177	0.000	0.005	7
Hyperacusis	0.004	-0.006	1.277	0.402	0.808	0.909	5
Somatic	0.003	-0.002	1.274	0.536	0.586	0.909	3
Jaw/neck problems	0.005	-0.002	1.274	0.715	0.543	0.909	4
Hearing loss	0.026	0.019	1.261	3.741	0.011	0.120	4
Laterality of Hearing loss	0.009	0.006	1.224	2.520	0.114	0.795	2
Frequency of Tinnitus	0.006	-0.013	1.235	0.306	0.909	0.909	6
Laterality of Tinnitus	0.007	-0.003	1.275	0.697	0.594	0.909	5
Fluctuation of Tinnitus	0.014	0.007	1.270	1.931	0.124	0.832	4
Duration of treatment	0.097	0.086	0.536	9.015	0.000	0.000	6
Sum	-	0.169	-	-	-	-	-

Table S9. summary of statistical models with the outcomes of the treatment ”Homeopathic Medication” as dependent variable

HBOT

	r.squared	adj.r.squared	sigma	statistic	p.value	Adjusted p.value	df
Gender	0.066	0.022	1.578	1.511	0.232	0.802	3
Age	0.110	-0.054	1.638	0.672	0.694	0.802	8
Onset	0.404	0.275	1.359	3.137	0.008	0.099	9
Noise reactivness	0.183	0.058	1.549	1.460	0.217	0.802	7
Hyperacusis	0.063	-0.029	1.619	0.688	0.604	0.802	5
Somatic	0.038	0.016	1.583	1.746	0.193	0.773	2
Jaw/neck problems	0.091	0.026	1.575	1.398	0.257	0.802	4
Hearing loss	0.069	0.026	1.575	1.590	0.216	0.802	3
Laterality of Hearing loss	0.159	0.130	1.485	5.466	0.027	0.265	2
Frequency of Tinnitus	0.084	-0.099	1.668	0.461	0.802	0.802	6
Laterality of Tinnitus	0.182	0.103	1.512	2.288	0.076	0.458	5
Fluctuation of Tinnitus	0.213	0.157	1.466	3.783	0.017	0.172	4
Duration of treatment	0.067	-0.050	0.606	0.573	0.720	0.802	6
Sum	-	0.581	-	-	-	-	-

Table S10. summary of statistical models with the outcomes of the treatment ”Hyperbaric Oxygen Therapy” as dependent variable

LLLT

	r.squared	adj.r.squared	sigma	statistic	p.value	Adjusted p.value	df
Gender	0.006	-0.026	1.844	0.177	0.838	0.988	3
Age	0.117	0.009	1.812	1.082	0.387	0.988	8
Onset	0.134	0.027	1.795	1.258	0.288	0.988	8
Noise reactivness	0.015	-0.086	1.898	0.151	0.988	0.988	7
Hyperacusis	0.067	0.005	1.816	1.078	0.375	0.988	5
Somatic	0.083	0.054	1.771	2.813	0.068	0.813	3
Jaw/neck problems	0.042	-0.006	1.826	0.883	0.455	0.988	4
Hearing loss	0.065	0.019	1.803	1.413	0.248	0.988	4
Laterality of Hearing loss	0.000	-0.022	1.622	0.014	0.907	0.988	2
Frequency of Tinnitus	0.078	-0.038	1.634	0.674	0.645	0.988	6
Laterality of Tinnitus	0.037	-0.010	1.830	0.780	0.510	0.988	4
Fluctuation of Tinnitus	0.032	-0.016	1.835	0.671	0.573	0.988	4
Duration of treatment	0.045	-0.035	0.768	0.562	0.729	0.988	6
Sum	-	-0.126	-	-	-	-	-

Table S11. summary of statistical models with the outcomes of the treatment "Low level laser therapy" as dependent variable

Sound Masker

	r.squared	adj.r.squared	sigma	statistic	p.value	Adjusted p.value	df
Gender	0.000	-0.004	1.870	0.043	0.958	0.960	3
Age	0.027	0.013	1.854	1.972	0.057	0.286	8
Onset	0.052	0.037	1.832	3.406	0.001	0.010	9
Noise reactivness	0.027	0.015	1.852	2.266	0.036	0.181	7
Hyperacusis	0.012	0.004	1.863	1.487	0.205	0.820	5
Somatic	0.020	0.016	1.852	5.011	0.007	0.062	3
Jaw/neck problems	0.022	0.016	1.851	3.704	0.012	0.094	4
Hearing loss	0.004	-0.002	1.869	0.599	0.616	0.960	4
Laterality of Hearing loss	0.017	0.014	1.904	6.140	0.014	0.109	2
Frequency of Tinnitus	0.003	-0.011	1.928	0.205	0.960	0.960	6
Laterality of Tinnitus	0.019	0.011	1.856	2.388	0.050	0.251	5
Fluctuation of Tinnitus	0.025	0.019	1.852	4.207	0.006	0.053	4
Duration of treatment	0.187	0.179	0.724	22.837	0.000	0.000	6
Sum	-	0.306	-	-	-	-	-

Table S12. summary of statistical models with the outcomes of the treatment "Sound Masker" as dependent variable

Neurofeedback and meditation

	r.squared	adj.r.squared	sigma	statistic	p.value	Adjusted p.value	df
Gender	0.010	-0.001	1.666	0.871	0.457	0.890	4
Age	0.019	-0.007	1.671	0.724	0.652	0.890	8
Onset	0.014	-0.017	1.679	0.450	0.890	0.890	9
Noise reactivness	0.017	-0.005	1.670	0.757	0.604	0.890	7
Hyperacusis	0.007	-0.008	1.672	0.444	0.776	0.890	5
Somatic	0.014	0.007	1.660	1.926	0.148	0.890	3
Jaw/neck problems	0.004	-0.007	1.671	0.377	0.770	0.890	4
Hearing loss	0.032	0.021	1.648	2.936	0.034	0.406	4
Laterality of Hearing loss	0.003	-0.004	1.603	0.439	0.509	0.890	2
Frequency of Tinnitus	0.038	0.007	1.595	1.227	0.299	0.890	6
Laterality of Tinnitus	0.007	-0.008	1.672	0.493	0.741	0.890	5
Fluctuation of Tinnitus	0.021	0.010	1.659	1.867	0.135	0.890	4
Duration of treatment	0.101	0.084	0.652	5.916	0.000	0.000	6
Sum	-	0.071	-	-	-	-	-

Table S13. summary of statistical models with the outcomes of the treatment "Neurofeedback and Meditation" as dependent variable

Neuromonics							
	r.squared	adj.r.squared	sigma	statistic	p.value	Adjusted p.value	df
Gender	0.008	-0.003	1.810	0.705	0.403	0.991	2
Age	0.058	-0.018	1.823	0.767	0.616	0.991	8
Onset	0.070	-0.017	1.823	0.803	0.601	0.991	9
Noise reactivness	0.105	0.044	1.767	1.720	0.126	0.847	7
Hyperacusis	0.057	0.015	1.794	1.357	0.255	0.968	5
Somatic	0.000	-0.022	1.827	0.009	0.991	0.991	3
Jaw/neck problems	0.017	-0.015	1.821	0.522	0.668	0.991	4
Hearing loss	0.026	-0.006	1.813	0.799	0.498	0.991	4
Laterality of Hearing loss	0.002	-0.013	1.933	0.147	0.703	0.991	2
Frequency of Tinnitus	0.082	0.011	1.911	1.151	0.343	0.991	6
Laterality of Tinnitus	0.057	0.015	1.793	1.364	0.253	0.968	5
Fluctuation of Tinnitus	0.014	-0.018	1.833	0.438	0.726	0.991	4
Duration of treatment	0.063	0.011	0.760	1.201	0.315	0.968	6
Sum	-	-0.017	-	-	-	-	-

Table S14. summary of statistical models with the outcomes of the treatment ”Neuromonics Treatment” as dependent variable

Notched Music							
	r.squared	adj.r.squared	sigma	statistic	p.value	Adjusted p.value	df
Gender	0.035	0.026	1.310	4.000	0.020	0.177	3
Age	0.012	-0.020	1.340	0.387	0.910	0.982	8
Onset	0.013	-0.024	1.343	0.353	0.944	0.982	9
Noise reactivness	0.032	0.005	1.324	1.202	0.307	0.982	7
Hyperacusis	0.013	-0.005	1.331	0.737	0.568	0.982	5
Somatic	0.000	-0.009	1.333	0.018	0.982	0.982	3
Jaw/neck problems	0.009	-0.004	1.330	0.697	0.555	0.982	4
Hearing loss	0.014	0.001	1.327	1.053	0.370	0.982	4
Laterality of Hearing loss	0.060	0.053	1.335	8.694	0.004	0.041	2
Frequency of Tinnitus	0.093	0.059	1.331	2.715	0.023	0.205	6
Laterality of Tinnitus	0.003	-0.015	1.337	0.187	0.945	0.982	5
Fluctuation of Tinnitus	0.057	0.044	1.301	4.396	0.005	0.055	4
Duration of treatment	0.120	0.099	0.543	5.904	0.000	0.001	6
Sum	-	0.212	-	-	-	-	-

Table S15. summary of statistical models with the outcomes of the treatment ”Notched Music” as dependent variable

Off label medication							
	r.squared	adj.r.squared	sigma	statistic	p.value	Adjusted p.value	df
Gender	0.002	-0.008	1.411	0.156	0.926	0.926	4
Age	0.020	-0.006	1.409	0.775	0.625	0.926	9
Onset	0.017	-0.009	1.411	0.672	0.716	0.926	9
Noise reactivness	0.012	-0.007	1.410	0.631	0.705	0.926	7
Hyperacusis	0.006	-0.007	1.410	0.494	0.740	0.926	5
Somatic	0.003	-0.004	1.407	0.448	0.639	0.926	3
Jaw/neck problems	0.022	0.013	1.396	2.333	0.074	0.741	4
Hearing loss	0.007	-0.003	1.407	0.735	0.532	0.926	4
Laterality of Hearing loss	0.012	0.007	1.373	2.364	0.126	0.864	2
Frequency of Tinnitus	0.019	-0.007	1.382	0.739	0.595	0.926	6
Laterality of Tinnitus	0.009	-0.004	1.408	0.686	0.602	0.926	5
Fluctuation of Tinnitus	0.028	0.019	1.392	2.992	0.031	0.374	4
Duration of treatment	0.165	0.151	0.548	12.082	0.000	0.000	6
Sum	-	0.137	-	-	-	-	-

Table S16. summary of statistical models with the outcomes of the treatment ”Off-Label Medication” as dependent variable

Psychiatrist

	r.squared	adj.r.squared	sigma	statistic	p.value	Adjusted p.value	df
Gender	0.013	0.003	2.033	1.285	0.280	0.874	4
Age	0.025	-0.002	2.038	0.941	0.483	0.874	9
Onset	0.063	0.037	1.998	2.414	0.015	0.139	9
Noise reactivness	0.038	0.018	2.017	1.921	0.077	0.541	7
Hyperacusis	0.045	0.032	2.004	3.418	0.009	0.085	5
Somatic	0.012	0.005	2.031	1.723	0.180	0.874	3
Jaw/neck problems	0.006	-0.004	2.041	0.567	0.637	0.874	4
Hearing loss	0.003	-0.007	2.043	0.335	0.800	0.874	4
Laterality of Hearing loss	0.041	0.036	2.018	7.601	0.006	0.064	2
Frequency of Tinnitus	0.010	-0.018	2.074	0.362	0.874	0.874	6
Laterality of Tinnitus	0.022	0.008	2.028	1.628	0.167	0.839	5
Fluctuation of Tinnitus	0.040	0.030	2.007	4.043	0.008	0.077	4
Duration of treatment	0.016	-0.001	0.762	0.945	0.452	0.874	6
Sum	-	0.136	-	-	-	-	-

Table S17. summary of statistical models with the outcomes of the treatment ”Psychiatrist” as dependent variable

Psychologist

	r.squared	adj.r.squared	sigma	statistic	p.value	Adjusted p.value	df
Gender	0.016	0.008	1.895	2.078	0.103	0.513	4
Age	0.035	0.014	1.889	1.694	0.098	0.499	9
Onset	0.006	-0.015	1.917	0.264	0.977	0.977	9
Noise reactivness	0.030	0.015	1.888	1.989	0.066	0.398	7
Hyperacusis	0.017	0.007	1.896	1.701	0.149	0.729	5
Somatic	0.002	-0.003	1.905	0.480	0.619	0.977	3
Jaw/neck problems	0.005	-0.002	1.905	0.690	0.559	0.977	4
Hearing loss	0.022	0.014	1.889	2.871	0.036	0.299	4
Laterality of Hearing loss	0.005	0.001	1.737	1.118	0.292	0.875	2
Frequency of Tinnitus	0.035	0.013	1.726	1.582	0.166	0.729	6
Laterality of Tinnitus	0.017	0.007	1.896	1.673	0.155	0.729	5
Fluctuation of Tinnitus	0.047	0.040	1.866	6.351	0.000	0.004	4
Duration of treatment	0.046	0.034	0.736	3.695	0.003	0.034	6
Sum	-	0.132	-	-	-	-	-

Table S18. summary of statistical models with the outcomes of the treatment ”Psychologist” as dependent variable

Retigabine

	r.squared	adj.r.squared	sigma	statistic	p.value	Adjusted p.value	df
Gender	0.106	0.089	2.359	6.070	0.017	0.206	2
Age	0.147	0.036	2.427	1.320	0.268	0.956	7
Onset	0.152	-0.002	2.474	0.988	0.459	0.956	9
Noise reactivness	0.173	0.085	2.364	1.964	0.102	0.709	6
Hyperacusis	0.007	-0.054	2.538	0.107	0.956	0.956	4
Somatic	0.083	0.047	2.413	2.275	0.113	0.709	3
Jaw/neck problems	0.046	-0.012	2.487	0.787	0.507	0.956	4
Hearing loss	0.079	0.023	2.443	1.401	0.254	0.956	4
Laterality of Hearing loss	0.000	-0.040	2.805	0.003	0.955	0.956	2
Frequency of Tinnitus	0.270	0.175	2.499	2.833	0.061	0.510	4
Laterality of Tinnitus	0.116	0.062	2.393	2.153	0.105	0.709	4
Fluctuation of Tinnitus	0.069	0.012	2.457	1.212	0.315	0.956	4
Duration of treatment	0.114	0.020	1.016	1.210	0.319	0.956	6
Sum	-	0.439	-	-	-	-	-

Table S19. summary of statistical models with the outcomes of the treatment ”Retigabine Administration” as dependent variable

Self Administered Sound Therapy

	r.squared	adj.r.squared	sigma	statistic	p.value	Adjusted p.value	df
Gender	0.010	0.008	1.423	5.375	0.001	0.010	4
Age	0.006	0.000	1.428	1.089	0.368	0.497	9
Onset	0.005	-0.000	1.429	0.922	0.497	0.497	9
Noise reactivness	0.049	0.046	1.396	13.487	0.000	0.000	7
Hyperacusis	0.011	0.008	1.423	4.253	0.002	0.018	5
Somatic	0.002	0.001	1.428	1.617	0.199	0.497	3
Jaw/neck problems	0.004	0.002	1.427	2.110	0.097	0.389	4
Hearing loss	0.004	0.002	1.427	1.955	0.119	0.462	4
Laterality of Hearing loss	0.004	0.003	1.443	3.640	0.057	0.340	2
Frequency of Tinnitus	0.007	0.002	1.443	1.375	0.231	0.497	6
Laterality of Tinnitus	0.004	0.001	1.428	1.435	0.220	0.497	5
Fluctuation of Tinnitus	0.015	0.013	1.421	7.700	0.000	0.000	4
Duration of treatment	0.099	0.096	0.653	34.203	0.000	0.000	6
Sum	-	0.182	-	-	-	-	-

Table S20. summary of statistical models with the outcomes of the treatment ”Self Administered Sound Therapy” as dependent variable

SoundCure

	r.squared	adj.r.squared	sigma	statistic	p.value	Adjusted p.value	df
Gender	0.004	-0.003	1.797	0.634	0.427	0.959	2
Age	0.042	-0.015	1.808	0.743	0.653	0.959	9
Onset	0.119	0.067	1.734	2.284	0.025	0.277	9
Noise reactivness	0.059	0.018	1.779	1.425	0.209	0.959	7
Hyperacusis	0.009	-0.020	1.813	0.305	0.874	0.959	5
Somatic	0.003	-0.011	1.805	0.228	0.796	0.959	3
Jaw/neck problems	0.031	0.010	1.786	1.488	0.221	0.959	4
Hearing loss	0.002	-0.019	1.812	0.101	0.959	0.959	4
Laterality of Hearing loss	0.020	0.009	1.800	1.811	0.182	0.959	2
Frequency of Tinnitus	0.206	0.159	1.658	4.370	0.001	0.017	6
Laterality of Tinnitus	0.025	-0.003	1.798	0.896	0.468	0.959	5
Fluctuation of Tinnitus	0.027	0.006	1.789	1.301	0.277	0.959	4
Duration of treatment	0.182	0.152	0.750	6.140	0.000	0.000	6
Sum	-	0.352	-	-	-	-	-

Table S21. summary of statistical models with the outcomes of the treatment ”SoundCure” as dependent variable

Steroids

	r.squared	adj.r.squared	sigma	statistic	p.value	Adjusted p.value	df
Gender	0.001	-0.008	1.681	0.063	0.979	0.979	4
Age	0.018	-0.002	1.676	0.890	0.515	0.979	8
Onset	0.020	-0.003	1.677	0.876	0.537	0.979	9
Noise reactivness	0.033	0.016	1.661	1.915	0.078	0.690	7
Hyperacusis	0.012	0.001	1.674	1.070	0.371	0.979	5
Somatic	0.010	0.004	1.671	1.749	0.176	0.878	3
Jaw/neck problems	0.016	0.007	1.668	1.856	0.137	0.806	4
Hearing loss	0.011	0.003	1.672	1.292	0.277	0.979	4
Laterality of Hearing loss	0.003	-0.002	1.701	0.613	0.435	0.979	2
Frequency of Tinnitus	0.007	-0.016	1.714	0.315	0.904	0.979	6
Laterality of Tinnitus	0.011	-0.000	1.675	0.963	0.428	0.979	5
Fluctuation of Tinnitus	0.013	0.005	1.670	1.543	0.203	0.895	4
Duration of treatment	0.017	0.002	0.776	1.167	0.325	0.979	6
Sum	-	0.006	-	-	-	-	-

Table S22. summary of statistical models with the outcomes of the treatment ”Steroids Administration” as dependent variable

Supplements and Herbal

	r.squared	adj.r.squared	sigma	statistic	p.value	Adjusted p.value	df
Gender	0.002	-0.001	1.178	0.603	0.613	0.640	4
Age	0.008	0.001	1.177	1.103	0.359	0.640	9
Onset	0.009	0.002	1.176	1.340	0.219	0.640	9
Noise reactivness	0.016	0.010	1.171	3.029	0.006	0.061	7
Hyperacusis	0.003	-0.001	1.178	0.804	0.523	0.640	5
Somatic	0.003	0.001	1.177	1.577	0.207	0.640	3
Jaw/neck problems	0.003	-0.000	1.178	0.968	0.407	0.640	4
Hearing loss	0.002	-0.001	1.178	0.692	0.557	0.640	4
Laterality of Hearing loss	0.020	0.019	1.165	14.271	0.000	0.002	2
Frequency of Tinnitus	0.010	0.002	1.175	1.340	0.245	0.640	6
Laterality of Tinnitus	0.002	-0.001	1.178	0.632	0.640	0.640	5
Fluctuation of Tinnitus	0.022	0.020	1.166	8.804	0.000	0.000	4
Duration of treatment	0.101	0.097	0.515	25.821	0.000	0.000	6
Sum	-	0.149	-	-	-	-	-

Table S23. summary of statistical models with the outcomes of the treatment ”Supplements and Herbal Administration” as dependent variable

Transcranial Magnetic Stimulation

	r.squared	adj.r.squared	sigma	statistic	p.value	Adjusted p.value	df
Gender	0.003	-0.020	2.376	0.130	0.720	0.832	2
Age	0.118	0.005	2.347	1.046	0.405	0.832	6
Onset	0.177	0.022	2.327	1.140	0.360	0.832	8
Noise reactivness	0.051	-0.070	2.434	0.421	0.832	0.832	6
Hyperacusis	0.274	0.221	2.076	5.167	0.004	0.047	4
Somatic	0.063	0.019	2.331	1.422	0.253	0.832	3
Jaw/neck problems	0.035	-0.036	2.395	0.490	0.691	0.832	4
Hearing loss	0.235	0.179	2.132	4.189	0.011	0.101	4
Laterality of Hearing loss	0.207	0.177	2.215	6.806	0.015	0.134	2
Frequency of Tinnitus	0.400	0.296	2.048	3.838	0.016	0.141	5
Laterality of Tinnitus	0.184	0.124	2.202	3.078	0.038	0.304	4
Fluctuation of Tinnitus	0.032	-0.039	2.398	0.455	0.715	0.832	4
Duration of treatment	0.161	0.054	0.736	1.499	0.212	0.832	6
Sum	-	0.931	-	-	-	-	-

Table S24. summary of statistical models with the outcomes of the treatment ”Transcranial Magnetic Stimulation” as dependent variable

Tinnitus Retraining Therapy

	r.squared	adj.r.squared	sigma	statistic	p.value	Adjusted p.value	df
Gender	0.008	-0.000	1.969	0.962	0.411	0.664	4
Age	0.065	0.047	1.922	3.607	0.001	0.010	8
Onset	0.040	0.019	1.950	1.888	0.061	0.425	9
Noise reactivness	0.035	0.019	1.951	2.173	0.045	0.315	7
Hyperacusis	0.016	0.005	1.964	1.491	0.204	0.613	5
Somatic	0.005	-0.000	1.969	0.934	0.394	0.664	3
Jaw/neck problems	0.034	0.026	1.943	4.285	0.005	0.055	4
Hearing loss	0.042	0.034	1.935	5.357	0.001	0.014	4
Laterality of Hearing loss	0.004	0.000	1.872	1.044	0.308	0.616	2
Frequency of Tinnitus	0.060	0.039	1.835	2.936	0.014	0.123	6
Laterality of Tinnitus	0.007	-0.004	1.973	0.598	0.664	0.664	5
Fluctuation of Tinnitus	0.011	0.003	1.966	1.348	0.259	0.616	4
Duration of treatment	0.144	0.132	0.781	12.225	0.000	0.000	6
Sum	-	0.319	-	-	-		-

Table S25. summary of statistical models with the outcomes of the treatment ”Tinnitus Retraining Therapy” as dependent variable
